# Sinapic Acid Derivatives as Potential Anti-Inflammatory Agents: Synthesis and Biological Evaluation

**Published:** 2017

**Authors:** Qiongyu Zhang, Jun-Xiao Hu, Xu Kui, Chao Liu, Hui Zhou, Xiaoxin Jiang, Leping Zeng

**Affiliations:** a *Department of Basic Medical Science, Yongzhou Vocational Technical College, Yong Zhou 425100, PR China. *; b *Department of Anatomy and Neurobiology, Biology Postdoctoral Workstation, Basic School of Medicine Central South University, Changsha, Hunan, 410013, China.*; c *Tumor Hospital Xiangya School of Medicine of Central South University, Changsha, Hunan, 410013, China. *; d *The First Affiliated Hospital, University of South China, Hengyang, Hunan, 421001, China.*

**Keywords:** Sinapic acid, Inflammation, Nuclear factor-kappa B, Interleukin

## Abstract

Transcription factor NF-κB and relevant cytokines IL-6 and IL-8 play a pivotal role in the pathogenesis of inflammation. Sinapic acid is a natural product and was demonstrated to possess anti-inflammatory activity. In this paper, we synthesized a series of sinapic acid derivatives and evaluated their anti-inflammatory effects. The result suggested that all of the targets compounds 7a-j inhibit NF-κB activation and decrease IL-6 and IL-8 expression in BEAS-2B cells. By our biological assays, we found that all of the prepared compounds displayed stronger anti-inflammatory activities than their precursor sinapic acid. Especially, compounds 7g and 7i, with electron-drawing groups (nitro and fluoro moieties) in the benzimidazole ring, exhibited remarkable anti-inflammation activity, which was even stronger than the reference drug dexamethasone.

## Introduction

Inflammation, characterized by redness, swelling, heat and pain, is close to many diseases such as cancer (1), arthritis (2), diabetes (3), diabetic nephropathy (4), cardiovascular diseases (5), ageing (6), Parkinsonꞌs disease (7), Alzheimer›s disease (8), and so on. In the pathogenesis of inflammation, nuclear factor-kappa B (NF-κB) plays a pivotal role to express many proinflammatory genes and results in the synthesis of cytokines and chemokines including interleukin IL-6, IL-8, RANTES, IL-11, and eotaxin (9, 10).

**Figure 1:**
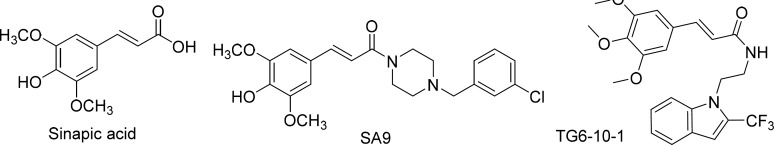
Typical structure of anti-inflammatory agents

In the discovery of anti-inflammatory agents, natural medicines play an important role. Sinapic acid (Figure 1), a natural product found in many medicinal herbs, such as *Brassica alba *(L.) Boiss and *Brassica juncea *(L.) coss. Pharmacological investigations have revealed sinapic acid can attenuate the carbon tetrachloride-induced acute hepatic injury (11), and ameliorate asthma (12) via antagonising inflammatory response. Its anti-inflammatory effect acts through suppressing iNOS, COX-2, and expressing the proinflammatory cytokines via inactivating NF-κB (13). However, its poor lipophilicity appeared to limit its further clinical application. In addition, its anti-inflammatory potency is far from satisfactory. Modification of sinapic acid to enhance its potency is imperative. It is well known to us that the introduction of heteroatoms or heterocycles into molecules frequently improves their physicochemical properties, thus increase their potency. Recently, a series of sinapic acid piperazine derivatives have been synthesized, of which, compound SA9 (Figure 1) showed significant inhibition of endothelial activation *in-vitro* and *in-vivo* via inflammatory pathway (14). More importantly, the TG-10-1 with indole moiety displayed anti-inflammatory and neuroprotective actions (15). Inspired by the aforementioned results, herein we have interest to report some novel sinapic acid derivatives with benzimidazoles pendant as potential anti-inflammatory agents. Also, their anti-inflammatory activities have been evaluated by inhibition of NF-κB and expression levels of IL-6 and IL-8.

## Experimental

*Chemicals and reagents*

Human tumor necrosis factor-α (TNF-α) was obtained from PeproTech (Rocky Hill, NJ). Dexamethasone was purchased from Sigma Chemical Co. (St. Louis, MO). The medium and reagents for cells culture were supplied by Life Technologies/Gibco, Thermo Fisher Scientific Inc. (Rockville, MD). The luciferase reporter assay system was purchased from Promega Co. (Madison, WI). The lipofectamine 2000 transfection reagent was obtained from Invitrogen life technologies, Thermo Fisher Scientific Inc. (Carlsbad, CA). Enzyme-linked immunosorbent assay kits (ELISA) were from Thermo Scientific Pierce Protein Biology Products, Thermo Fisher Scientific Inc. (Rockford, IL). The other solvents employed in this study were analytical purity grade.

**Figure 2:**
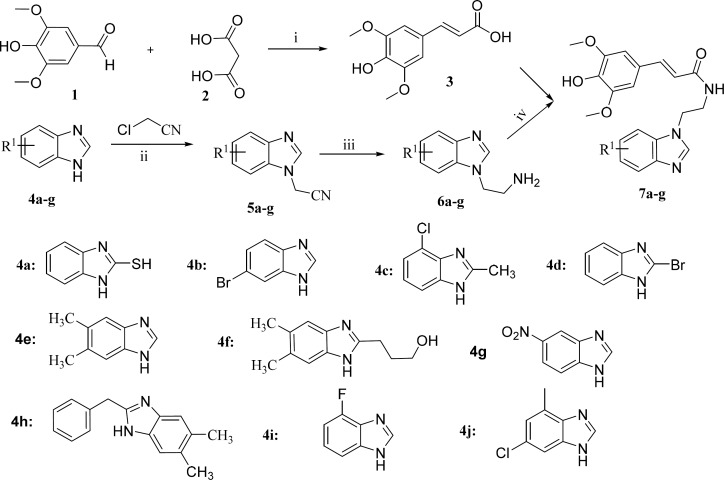
Inhibition effects of the target compounds 7a-j on NF-κB in BEAS-2B cells stimulated by TNF-α, ^*^ *p *< 0.05 *vs* control group, ^#^ *p *< 0.05 *vs* model group

**Figure 3:**
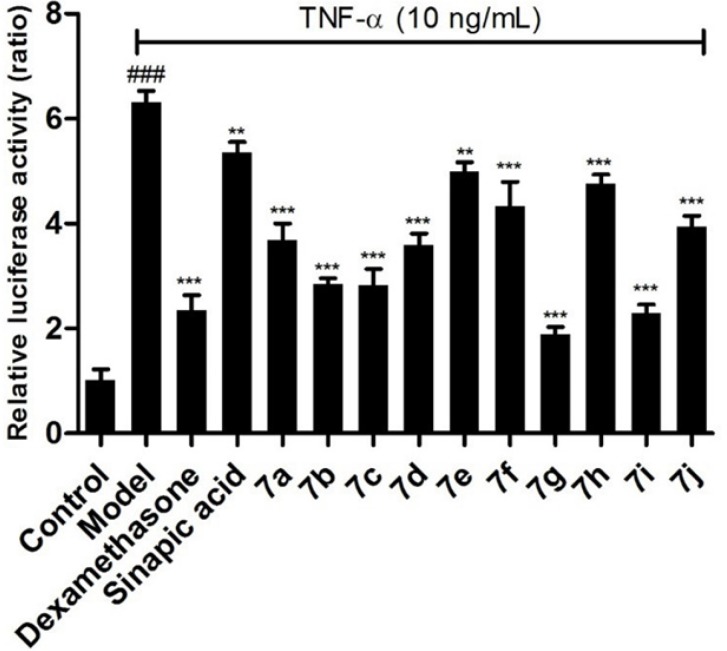
Effects of the target compounds 7a-j on IL-6 expression in supernatant of BEAS-2B cells stimulated by TNF-α, ^*^ *p *< 0.05 *vs* control group, ^#^ *p *< 0.05 *vs* model group

**Figure 4.**
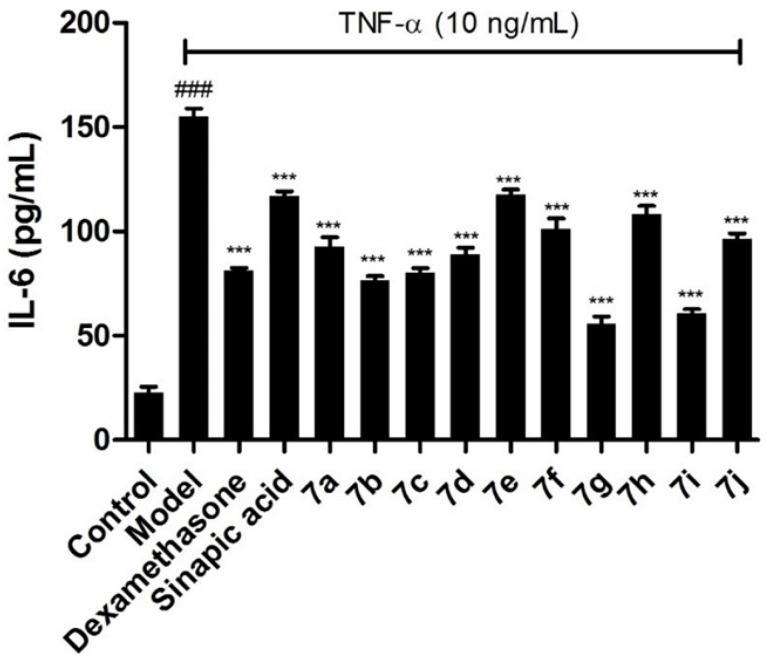
Effects of the target compounds 7a-j on IL-8 expression in supernatant of BEAS-2B cells stimulated by TNF-α, ^*^ *p *< 0.05 *vs* control group, ^#^ *p *< 0.05 *vs* model group

**Scheme 1:**
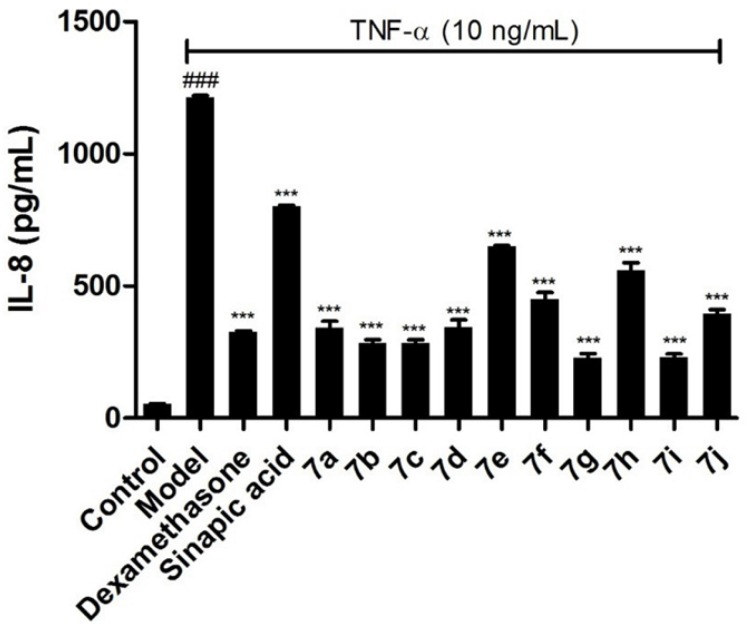
Synthetic routes for the sinapic acid analogs. Reagents and conditions: (i) Pyridine, piperidine, 22 h, rt; (ii) CH_3_CN, K_2_CO_3_, 70 °C, 12 h; (iii) THF, LiAlH_4_, 0 °C, 2 h; (iv) Oxalic dichloride, DMF, 0 °C, 5 h

TLC analysis was done using pre**-**coated silica gel plates. ^1^H NMR spectra were recorded on a Bruker AV 300 spectrometer using TMS as an internal standard. The chemical shifts were reported in parts per million (ppm), the signals were described as singlet (s), doublet (d), triplet (t), as well as multiplet (m). The high**-**resolution mass spectra (HRMS) were recorded on an IonSpec FT**-**ICR mass spectrometer with ESI resource. All other chemicals and solvents were commercially available, and were used without further purification.

*Cell culture*

BEAS-2B cells, derived from human bronchial epithelial cells, were obtained from American type culture collection (Rockville, MD) and cultured using DMEM/F12 medium with 10% fetal bovine serum in 96-well plates and placed in a humid, 5% CO_2_ atmosphere at 37 °C.

*Transfection*

BEAS-2B cells were co-transfected with NF-κB luciferase reporter plasmid pGL4.32 and Renilla luciferase reporter vector plasmid pRL-TK at 100 and 9.6 ng per well respectively. Transfection was performed for 24 h using lipofectamine 2000 according to the manufacturer’s instructions. The medium was replaced with fresh serum-free medium. The Cells cells were pretreated with dexamethasone (0.01 mM) or target compounds (0.1 mM) before the addition of TNF-α (10 ng/mL).

*Luciferase reporter assay for inhibition of NF-κB*

After stimulation, BEAS-2B cells were washed, lysed, and assayed by luciferase activity using luciferase reporter assay system according to the manufacturer’s instructions. Relative luciferase activity was obtained by normalizing the firefly luciferase activity against that of the internal control (Renilla luciferase).

*Measurements of IL-6 and IL-8*

Commercial ELISA kits were employed to measure the release of IL-6 and IL-8 in the supernatants of BEAS-2B cells after the administration of dexamethasone (0.01 mM) or target compounds (0.1 mM) and stimulation of TNF-α. The absorbance of each sample was determined at 450 nm by a Bio-Rad model 680 microplate reader. Levels of IL-6 and IL-8 were measured from standard curves and expressed as picograms per milliliter.

*Statistical analysis*

All values are expressed as the means ± standard deviation. SPSS software was employed for the statistical analysis. Significant differences of experimental data from different groups were compared by one way analysis of variance (one-way ANOVA) for multiple comparisons and Student’s *t-*test for single comparisons. And, and the level *p* < 0.05 was considered as statistical significance.

*Synthesis of the intermediates and target compounds*

*Synthesis of (E)-3-(4-hydroxy-3, 5-dimethoxyphenyl) acrylic acid (3)*

To pyridine (6.4 mL) was added commercial syringaldehyde (2.12 g, 11.3 mmol), malonic acid (2.43 g, 22.6 mmol), and piperidine (0.22 mL, 2.3 mmol). After 22 h of stirring at room temperature, the reaction was quenched by addition of 7.5 mL of concentrated aqueous HCl solution. After addition of 94 mL water, the formed precipitate was filtered and dried to get compound 3 (1.79 g, Yield: 71%). 

*Synthesis of intermediates (5)*

Benzimidazole 4a (1.51 g, 10 mmol) and potassium carbonate (1.67 g, 12 mmol) were mixture together in acetonitrile (20 mL) for 0.5 h. Then, 2-chloroacetonitrile (0.61 g, 11 mmol) was added dropwise, and the mixture was stirred at 70 °C. After the reaction was completed, the mixture was cooled to room temperature, then the solvent was evaporated under vacuum, and water (30 mL) was added. The resulting mixture was extracted with dichlormethane (3 × 20 mL), then the combined organic phase was dried over anhydrous sodium sulfate and subsequently the solvent was evaporated under reduced pressure. The residue was purified by silica gel column chromatography (eluent, petroleum/ethyl acetate, 2/1, V/V) to give the desired compound 5a (1.13 g, Yield: 60%).

*2-(6-bromo-1H-benzo[d]imidazol-1-yl) acetonitrile (5b)*

Compound 5b was prepared according to the procedure depicted for compound 5a, starting from compound 4b (1.96 g, 10 mmol), potassium carbonate (1.67 g, 12 mmol) and 2-chloroacetonitrile (0.61 g, 11 mmol). The product 5b (1.51 g) was obtained as white solid. Yield: 64%; ^1^H NMR (300 MHz, CDCl_3_): δ 8.05 (s, 1H), 7.89 (m, 1H), 7.43 (m, 1H), 7.32 (s, 1H), 5.23 (s, 2H); HRMS (ESI) calcd. For C_9_H_7_N_3_Br [M+H]^+^, 237.0760; found, 237.0762. 

*2-(4-chloro-2-methyl-1H-benzo[d]imidazol-1-yl) acetonitrile (5c)*

Compound 5c was prepared according to the procedure depicted for compound 5a, starting from compound 4c (1.66 g, 10 mmol), potassium carbonate (1.67 g, 12 mmol) and 2-chloroacetonitrile (0.61 g, 11 mmol). The product 5b (1.16 g) was obtained as white solid. Yield: 57%; ^1^H NMR (300 MHz, CDCl_3_): δ 7.47 (m, 2H), 7.41 (m, 1H), 5.25 (s, 2H), 2.37 (s, 3H); HRMS (ESI) calcd. For C_10_H_9_N_3_Cl [M+H]^+^, 206.0485; found, 206.0479. 

*2-(2-bromo-1H-benzo[d]imidazol-1-yl) acetonitrile (5d)*

Compound 5d was prepared according to the procedure depicted for compound 5a, starting from compound 4d (1.97 g, 10 mmol), potassium carbonate (1.67 g, 12 mmol), and 2-chloroacetonitrile (0.61 g, 11 mmol). The product 5d (1.13 g) was obtained as white solid. Yield: 48%; ^1^H NMR (300 MHz, CDCl_3_): δ 7.87 (m, 1H), 7.46 (m, 2H), 7.42 (m, 1H), 5.26 (s, 2H); HRMS (ESI) calcd. For C_9_H_7_N_3_Br [M+H]^+^, 237.0760; found, 237.0756.

*2-(2-(3-hydroxypropyl)-5-methyl-1H-benzo[d]imidazol-1-yl) acetonitrile (5f)*

Compound 5f was prepared according to the procedure depicted for compound 5a, starting from compound 4f (2.04 g, 10 mmol), potassium carbonate (1.67 g, 12 mmol) and 2-chloroacetonitrile (0.61 g, 11 mmol). The product 5d (0.99 g) was obtained as white solid. Yield: 41.0%; ^1^H NMR (300 MHz, CDCl_3_): δ 7.89 (s, 1H), 7.33 (s, 1H), 5.25 (s, 2H), 3.77 (m, 2H), 2.62 (m, 2H), 2.35 (m, 2H), 2.30 (s, 6H); HRMS (ESI) calcd. For C_14_H_18_N_3_O [M+H]^+^, 244.3180; found, 244.3185.

*2-(5-nitro-1H-benzo[d]imidazol-1-yl) acetonitrile (5g)*

Compound 5g was prepared according to the procedure depicted for compound 5a, starting from compound 4g (1.63 g, 10 mmol), potassium carbonate (1.67 g, 12 mmol) and 2-chloroacetonitrile (0.61 g, 11 mmol). The product 5g (1.01 g) was obtained as white solid. Yield: 50.0%; ^1^H NMR (300 MHz, CDCl_3_): δ 8.32 (s, 1H), 8.13 (m, 1H), 7.64 (m, 1H), 7.39 (s, 1H), 5.24 (s, 2H); HRMS (ESI) calcd. For C_9_H_7_N_4_O_2_ [M+H]^+^, 203.0569; found, 203.0572. 

*2-(4-fluoro-1H-benzo[d]imidazol-1-yl) acetonitrile (5i)*

Compound 5i was prepared according to the procedure depicted for compound 5a, starting from compound 4i (1.36 g, 10 mmol), potassium carbonate (1.67 g, 12 mmol) and 2-chloroacetonitrile (0.61 g, 11 mmol). The product 5g (1.20 g) was obtained as white solid. Yield: 68.6%; ^1^H NMR (300 MHz, CDCl_3_): δ 8.29 (m, 1H), 8.06 (m, 1H), 7.61 (m, 1H), 7.40 (s, 1H), 5.22 (s, 2H); HRMS (ESI) calcd. For C_9_H_7_N_3_F [M+H]^+^, 176.0624; found, 176.0629.

*2-(6-chloro-4-methyl-1H-benzo[d]imidazol-1-yl) acetonitrile (5j)*

Compound 5j was prepared according to the procedure depicted for compound 5a, starting from compound 4j (1.66 g, 10 mmol), potassium carbonate (1.67 g, 12 mmol) and 2-chloroacetonitrile (0.61 g, 11 mmol). The product 5j (1.60 g) was obtained as white solid. Yield: 80.0%; ^1^H NMR (300 MHz, CDCl_3_): δ 8.22 (s, 1H), 7.40 (s, 1H), 7.22 (s, 1H), 5.22 (s, 2H), 2.02 (s, 3H); HRMS (ESI) calcd. For C_10_H_9_N_3_Cl [M+H]^+^, 206.0485; found, 206.0486.

*Synthesis of intermediates (6)*

To a solution of 5a (0.95 g, 5 mmol) in THF was added LiAlH_4_ (0.76 g, 10 mmol, 2.0 equiv), dropwise at 0 °C, and the resulting reaction mixture was brought to room temperature overnight. Methanol (2 mL) was slowly added to quench the reaction at 0 °C, followed by 1 N NaOH (3 mL) at room temperature. The product was extracted with ethyl ether (30 mL × 3). Organics were washed with water, brine and dried over Na_2_SO_4_ and concentrated. The crude mass was subjected to silica gel chromatography, eluting with 0−5% methanol in dichloromethane to provide 6a (0.81 g, Yield: 84.1%).

*2-(6-bromo-1H-benzo[d]imidazol-1-yl) ethan-1-amine (6b)*

Compound 6b was prepared according to the procedure depicted for compound 6a, starting from compound 5b (1.2 g, 5 mmol) and LiAlH_4_ (0.76 g, 10 mmol, 2.0 equiv). The product 6b (1.1 g) was obtained as white solid. Yield: 95.2%. ^1^H NMR (300 MHz, CDCl_3_): δ 8.02 (s, 1H), 7.86 (m, 1H), 7.41 (m, 1H), 7.31 (s, 1H), 4.40 (m, 2H), 3.08 (m, 2H), 1.10 (br, 2H); HRMS (ESI) calcd. For C_9_H_11_N_3_Br [M+H]^+^, 241.1120; found, 241.1123. 

*2-(4-chloro-2-methyl-1H-benzo[d]imidazol-1-yl) ethan-1-amine (6c)*

Compound 6c was prepared according to the procedure depicted for compound 6a, starting from compound 5c (1.0 g, 5 mmol) and LiAlH_4_ (0.76 g, 10 mmol, 2.0 equiv). The product 6c (0.94 g) was obtained as desired compound. Yield: 90.0%. ^1^H NMR (300 MHz, CDCl_3_): δ 7.46 (m, 2H), 7.40 (m, 1H), 4.43 (m, 2H), 3.06 (m, 2H), 2.37 (s, 3H), 1.14 (br, 2H); HRMS (ESI) calcd. For C_10_H_13_N_3_Cl [M+H]^+^, 210.6850; found, 210.6854.

*2-(2-bromo-1H-benzo[d]imidazol-1-yl) ethan-1-amine (6d)*

Compound 6d was prepared according to the procedure depicted for compound 6a, starting from compound 5d (1.2 g, 5 mmol) and LiAlH_4_ (0.76 g, 10 mmol, 2.0 equiv). The product 6d (1.2 g) was obtained as desired compound. Yield: 93.0%. ^1^H NMR (300 MHz, CDCl_3_): δ 7.89 (m, 1H), 7.45 (m, 2H), 7.41 (m, 1H), 4.45 (m, 2H), 3.08 (m, 2H), 1.14 (br, 2H); HRMS (ESI) calcd. For C_9_H_11_N_3_Br [M+H]^+^, 241.1120; found, 241.1118.

*2-(5, 6-dimethyl-1H-benzo[d]imidazol-1-yl) ethan-1-amine (6e)*

Compound 6e was prepared according to the procedure depicted for compound 6a, starting from compound 5e (0.93 g, 5 mmol) and LiAlH_4_ (0.76 g, 10 mmol, 2.0 equiv). The product 6e (0.83 g) was obtained as desired compound. Yield: 87.0%. 

*3-(1-(2-aminoethyl)-5, 6-dimethyl-1H-benzo[d]imidazol-2-yl) propan-1-ol (6f)*

Compound 6f was prepared according to the procedure depicted for compound 6a, starting from compound 5f (1.25 g, 5 mmol) and LiAlH_4_ (0.76 g, 10 mmol, 2.0 equiv). The product 6f (0.96 g) was obtained as desired compound. Yield: 77.1%. ^1^H NMR (300 MHz, CDCl_3_): δ 7.88 (s, 1H), 7.36 (s, 1H), 4.44 (m, 2H), 3.76 (m, 2H), 3.06 (m, 2H), 2.62 (m, 2H), 2.31 (m, 2H), 2.28 (s, 6H), 1.14 (br, 2H); HRMS (ESI) calcd. For C_14_H_22_N_3_O [M+H]^+^, 248.3500; found, 248.3503. 

*2-(5-nitro-1H-benzo[d]imidazol-1-yl) ethan-1-amine (6g)*

Compound 6g was prepared according to the procedure depicted for compound 6a, starting from compound 5g (1.00 g, 5 mmol) and LiAlH_4_ (0.76 g, 10 mmol, 2.0 equiv). The product 6g (1.32 g) was obtained as desired compound. Yield: 64.0%. ^1^H NMR (300 MHz, CDCl_3_): δ 8.33 (s, 1H), 8.09 (m, 1H), 7.67 (m, 1H), 7.40 (s, 1H), 4.43 (m, 2H), 3.10 (m, 2H), 1.15 (br, 2H); HRMS (ESI) calcd. For C_9_H_11_N_4_O_2_ [M+H]^+^, 207.0882; found, 207.0884.

*2-(2-benzyl-5, 6-dimethyl-1H-benzo[d]imidazol-1-yl) ethan-1-amine (6h)*

Compound 6g was prepared according to the procedure depicted for compound 6a, starting from compound 5g (1.38 g, 5 mmol) and LiAlH_4_ (0.76 g, 10 mmol, 2.0 equiv). The product 6f (1.20 g) was obtained as desired compound. Yield: 86%. 

*2-(4-fluoro-1H-benzo[d]imidazol-1-yl) ethan-1-amine (6i)*

Compound 6i was prepared according to the procedure depicted for compound 6a, starting from compound 5i (0.85 g, 5 mmol) and LiAlH_4_ (0.76 g, 10 mmol, 2.0 equiv). The product 6i (1.13 g) was obtained as desired compound. Yield: 62.8%. ^1^H NMR (300 MHz, CDCl_3_): ^1^H NMR (300 MHz, CDCl_3_): δ 8.30 (m, 1H), 8.09 (m, 1H), 7.59 (m, 1H), 7.39 (s, 1H), 4.41 (m, 2H), 3.11 (m, 2H), 1.12 (br, 2H); HRMS (ESI) calcd. For C_9_H_11_N_3_F [M+H]^+^, 180.0937; found, 180.0942.

*2-(6-chloro-4-methyl-1H-benzo[d]imidazol-1-yl) ethan-1-amine (6j)*

Compound 6j was prepared according to the procedure depicted for compound 6a, starting from compound 5j (1.02 g, 5 mmol) and LiAlH_4_ (0.76 g, 10 mmol, 2.0 equiv). The product 6i (0.85 g) was obtained as desired compound. Yield: 40.7%. ^1^H NMR (300 MHz, CDCl_3_): δ 8.21 (s, 1H), 7.43 (s, 1H), 7.21 (s, 1H), 4.42 (m, 2H), 3.08 (m, 2H), 2.03 (s, 3H), 1.13 (br, 2H); HRMS (ESI) calcd. For C_10_H_13_N_3_Cl [M+H]^+^, 210.0798; found, 210.0794.

*Synthesis of the target compounds 7a-j*

To a solution of 3 (0.9 g, 4.0 mmol) in dichloromethane (15 mL) were added oxalic dichloride (0.64 g, 5.0 mmol) dropwise, the mixture was stirred at ice bath. One drop of *N, N*-dimethylformamide was added. Then, compound 6a (0.77 g, 4.0 mmol) in dichloromethane was added. The resulting reaction mixture was stirred at room temperature for 8 h. The reaction was quenched with water (10 mL), and the product was extracted with ethyl acetate (20 mL × 3). Organics were washed with water (20 mL), brine solution (20 mL) and dried over Na_2_SO_4_. The crude product was purified by silica gel chromatography, eluting with 0−35% ethyl acetate in hexane to provide the target compound 7a (1.32 mg, Yield: 83%). ^1^H NMR (300 MHz, CDCl_3_): δ 7.63 (m, 2H), 7.28 (m, 2H), 6.89 (s, 1H), 6.63 (s, 1H), 6.41 (m, 1H), 6.25 (m, 1H), 4.43 (m, 2H), 3.79 (s, 3H), 3.77 (s, 6H), 3.70 (m, 2H), 2.6 (br, 1H); HRMS (ESI) calcd. For C_20_H_22_N_3_O_4_S [M+H]^+^, 400.4714; found, 400.4715.

*(E)-N-(2-(7-bromo-1H-benzo[d]imidazol-1-yl) ethyl)-3-(4-hydroxy-3, 5-dimethoxyphenyl) acrylamide (7b)*

Compound 7b was prepared according to the procedure depicted for compound 7a, starting from compound 3 (0.9 g, 4.0 mmol) and compound 6b (0.96 g, 4.0 mmol). The product 7b (1.30 g) was obtained as desired compound. Yield: 73%. ^1^H NMR (300 MHz, CDCl_3_): δ 8.01 (s, 1H), 7.83 (m, 1H), 7.38 (m, 1H), 7.32 (s, 1H), 6.89 (s, 1H), 6.63 (s, 1H), 6.41 (m, 1H), 6.25 (m, 1H), 4.42 (m, 2H), 3.77 (s, 3H), 3.74 (s, 3H), 3.68 (m, 2H); HRMS (ESI) calcd. For C_20_H_21_N_3_O_4_Br [M+H]^+^, 447.3024; found, 447.3021. 

*(E)-N-(2-(4-chloro-2-methyl-1H-benzo[d]imidazol-1-yl)ethyl)-3-(4-hydroxy-3, 5-dimethoxyphenyl) acrylamide (7c)*

Compound 7c was prepared according to the procedure depicted for compound 7a, starting from compound 3 (0.9 g, 4.0 mmol) and compound 6c (0.84 g, 4.0 mmol). The product 7c (1.30 g) was obtained as desired compound. Yield: 78%. ^1^H NMR (300 MHz, CDCl_3_): δ 7.45 (m, 2H), 7.38 (m, 1H), 6.89 (s, 1H), 6.63 (s, 1H), 6.41 (m, 1H), 6.25 (m, 1H), 4.45 (m, 2H), 3.77 (s, 3H), 3.74 (s, 3H), 3.63 (m, 2H), 2.37 (s, 3H); HRMS (ESI) calcd. For C_21_H_23_N_3_O_4_Cl [M+H]^+^]^ +^, 417.8780; found, 417.8782. 

*(E)-N-(2-(2-bromo-1H-benzo[d]imidazol-1-yl) ethyl)-3-(4-hydroxy-3, 5-dimethoxyphenyl) acrylamide (7d)*

Compound 7d was prepared according to the procedure depicted for compound 7a, starting from compound 3 (0.9 g, 4.0 mmol) and compound 6d (0.96 g, 4.0 mmol). The product 7d (1.23 g) was obtained as desired compound. Yield: 69%. ^1^H NMR (300 MHz, CDCl_3_): δ 7.88 (m, 1H), 7.43 (m, 2H), 7.37 (m, 1H), 6.88 (s, 1H), 6.62 (s, 1H), 6.40 (m, 1H), 6.27 (m, 1H), 4.45 (m, 2H), 3.76 (s, 3H), 3.72 (s, 6H), 3.63 (m, 2H), 2.37 (s, 3H); HRMS (ESI) calcd. For C_20_H_21_N_3_O_4_Br [M+H]^+^, 447.3024; found, 447.3021. 

*(E)-N-(2-(5,6-dimethyl-1H-benzo[d]imidazol-1-yl)ethyl)-3-(4-hydroxy-3,5-dimethoxyphenyl) acrylamide (7e)*

Compound 7e was prepared according to the procedure depicted for compound 7a, starting from compound 3 (0.9 g, 4.0 mmol) and compound 6e (0.76 g, 4.0 mmol). The product 7e (1.40 g) was obtained as desired compound. Yield: 89%. ^1^H NMR (300 MHz, CDCl_3_): δ 7.72 (s, 1H), 7.36 (s, 1H), 7.16 (s, 1H), 6.88 (s, 1H), 6.62 (s, 1H), 6.40 (m, 1H), 6.27 (m, 1H), 4.45 (m, 2H), 3.76 (s, 3H), 3.72 (s, 6H), 3.63 (m, 2H), 2.18 (s, 3H), 2.15 (s, 3H); HRMS (ESI) calcd. For C_22_H_26_N_3_O_4_ [M+H]^+^]^ +^, 396.4595; found, 396.4597. 

*(E)-3-(4-hydroxy-3, 5-dimethoxyphenyl)-N-(2-(2-(3-hydroxypropyl)-5, 6-dimethyl-1H-benzo[d]imidazol-1-yl) ethyl) acrylamide (7f)*

Compound 7f was prepared according to the procedure depicted for compound 7a, starting from compound 3 (0.9 g, 4.0 mmol) and compound 6f (0.81 g, 4.0 mmol). The product 7f (0.69 g) was obtained as desired compound. Yield: 38%. ^1^H NMR (300 MHz, CDCl_3_): δ 7.88 (s, 1H), 7.36 (s, 1H), 6.87 (s, 1H), 6.63 (s, 1H), 6.42 (m, 1H), 6.28 (m, 1H), 4.45 (m, 2H), 3.79 (m, 2H), 3.76 (s, 3H), 3.72 (s, 6H), 3.63 (m, 2H), 2.62 (m, 2H), 2.31 (m, 2H), 2.28 (s, 6H); HRMS (ESI) calcd. For C_25_H_32_N_3_O_5_ [M+H]^+^]^ +^, 454.5387; found, 454.5386. 

*(E)-3-(4-hydroxy-3, 5-dimethoxyphenyl)-N-(2-(5-nitro-1H benzo[d]imidazol-1-yl) ethyl) acrylamide (7g)*

Compound 7g was prepared according to the procedure depicted for compound 7a, starting from compound 3 (0.9 g, 4.0 mmol) and compound 6h (0.82 g, 4.0 mmol). The product 7h (0.7 g) was obtained as desired compound. Yield: 42.4%. ^1^H NMR (300 MHz, CDCl_3_): δ 8.32 (s, 1H), 8.10 (s, 1H), 7.70 (s, 1H), 7.38 (s, 1H), 6.89 (s, 1H), 6.63 (s, 1H), 6.41 (m, 1H), 6.25 (m, 1H), 4.45 (m, 2H), 3.77 (s, 3H), 3.74 (s, 3H), 3.63 (m, 2H); HRMS (ESI) calcd. For C_20_H_21_N_4_O_6_ [M+H]^+^, 413.1461; found, 413.1464.

*(E)-N-(2-(2-benzyl-5, 6-dimethyl-1H-benzo[d]imidazol-1-yl) ethyl)-3-(4-hydroxy-3, 5-dimethoxyphenyl) acrylamide (7h)*

Compound 7h was prepared according to the procedure depicted for compound 7a, starting from compound 3 (0.9 g, 4.0 mmol) and compound 6h (1.12 g, 4.0 mmol). The product 7h (1.63 g) was obtained as desired compound. Yield: 84%. ^1^H NMR (300 MHz, CDCl_3_): δ 7.88 (s, 1H), 7.36 (s, 1H), 7.05 (m, 5H), 6.87 (s, 1H), 6.63 (s, 1H), 6.42 (m, 1H), 6.28 (m, 1H), 4.45 (m, 2H), 4.25 (s, 2H), 3.79 (m, 2H), 3.76 (s, 3H), 3.72 (s, 6H), 2.28 (s, 6H); HRMS (ESI) calcd. For C_29_H_32_N_3_O_4_ [M+H]^+^, 486.5821; found, 486.5823. 

*(E)-N-(2-(5-fluoro-1H-benzo[d]imidazol-1-yl) ethyl)-3-(4-hydroxy-3, 5-dimethoxy phenyl) acrylamide (7i)*

Compound 7g was prepared according to the procedure depicted for compound 7a, starting from compound 3 (0.9 g, 4.0 mmol) and compound 6g (0.72 g, 4.0 mmol). The product 7g (0.78 g) was obtained as desired compound. Yield: 50.6%. ^1^H NMR (300 MHz, CDCl_3_): δ 8.28 (m, 1H), 8.07 (m, 1H), 7. 69 (s, 1H), 7.39 (s, 2H), 6.88 (s, 1H), 6.63 (s, 1H), 6.41 (m, 1H), 6.23 (m, 1H), 4.44 (m, 2H), 3.79 (s, 3H), 3.71 (s, 3H), 3.62 (m, 2H); HRMS (ESI) calcd. For C_20_H_21_FN_3_O_4_ [M+H]^+^, 386.1516; found, 386.1519. 

*(E)-N-(2-(5-chloro-7-methyl-1H-benzo[d]imidazol-1-yl) ethyl)-3-(4-hydroxy-3, 5-dimethoxyphenyl) acrylamide (7j)*

Compound 7j was prepared according to the procedure depicted for compound 7a, starting from compound 3 (0.9 g, 4.0 mmol) and compound 6j (0.84 g, 4.0 mmol). The product 7j (1.0 g) was obtained as desired compound. Yield: 60.2%. ^1^H NMR (300 MHz, CDCl_3_): δ 8.23 (s, 1H), 7.41 (s, 1H), 7.22 (s, 1H), 6.88 (s, 1H), 6.62 (s, 1H), 6.41 (m, 1H), 6.22 (m, 1H), 4.44 (m, 2H), 3.79 (s, 3H), 3.71 (s, 3H), 3.62 (m, 2H), 2.03 (s, 3H); HRMS (ESI) calcd. For C_21_H_23_ClN_3_O_4_ [M+H]^+^, 416.1377; found, 416.1376.

## Results and Discussion

*Chemistry*

The synthetic route of the target sinapic acid derivatives was outlined in Scheme 1. The desired compounds were synthesized via multistep reactions from syringaldehyde and benzimidazoles. The intermediates 3 could be prepared in yield of 71% by knoevenagel-doebner reaction (16). Then the reaction of chloroacetonitrile with different types of benzimidazoles 4a-j yielded compounds 5a-j, which were subjected to reduction to afford their corresponding primary amines 6a-j in yields ranging from 40.7% to 95%. Finally, the target sinapic acid derivatives 7a-j were conveniently synthesized by the coupling of sinapic acid 3 and compounds 6a-j in the presence of oxalic dichloride at 0 °C for 5 h. Finally, all of the new compounds were characterized by ^1^H NMR and HRMS spectra.

*Inhibitory effects on NF-κB*

To investigate the anti-inflammatory activity of the target compounds, the luciferase reporter assay system has been employed to evaluate the inhibitory effects on NF-κB. Comparing with the model group, all of the prepared compounds gave stronger inhibitory effects. Compounds 7a, 7e, 7f, 7h, and 7j, with electron-donating groups in benzimidazole group, displayed relatively weaker activities than the clinic drug dexamethasone (Figure 2). To our surprise, compounds 7g and 7i with electron-drawing groups (nitro and fluoro moieties) showed the best inhibitory effects on the NF-κB in BEAS-2B cells stimulated by TNF-α. This suggests that the electron-drawing groups are beneficial to this series compounds to exert their inhibitory activity.

*Levels of IL-6 and IL-8*

To further confirm the inhibitory effects of the target compounds on NF-κB, the levels of IL-6 and IL-8 in the supernatants of BEAS-2B cells treated with TNF-α were measured by ELISA kits. As shown in Figures 3 and 4, all of the compounds could decrease the synthesis of IL-6 and IL-8 significantly in BEAS-2B cells. Comparing with sinapic acid, all of the compounds exhibited improved inhibitory effects on the synthesis of IL-6, except for compounds 7e and 7h. But for the IL-8, all of the compounds showed better inhibitory potencies than the precursor sinapic acid. In addition, compound 7g and 7i exhibited the most potent to reduce the synthesis of IL-8 and IL-6. From this hit, we could figure out that the introduction of the benzimidazole group could improve the activity of the sinapic acids and also the electron-drawing groups are in favor of enhancing the activity of the sinapic acid derivatives.

## Conclusions

In conclusion, a novel series of benzimidazole sinapic acid hybrids were synthesized via an easy, convenient, and efficient synthetic route starting from commercially available syringaldehyde and benzimidazoles in good yields, and all the new compounds were characterized by ^1^H NMR and HRMS spectra. The* in-vitro* anti-inflammatory activity was evaluated by the luciferase reporter assay system. The results suggest that the compounds 7g and 7i with electron-drawing groups showed the best inhibitory effects on the NF-κB in BEAS-2B cells stimulated by TNF-α. The ELISA assay revealed that compounds 7g and 7i could decrease the expression of IL-6 and IL-8 significantly, and their inhibitory effects were much better than their precursor sinapic acid. 
